# Investigation of Avian Influenza H5N6 Virus-like Particles as a Broad-Spectrum Vaccine Candidate against H5Nx Viruses

**DOI:** 10.3390/v14050925

**Published:** 2022-04-28

**Authors:** Yu-Hsuan Yang, Ching-Hui Tai, Dayna Cheng, Ya-Fang Wang, Jen-Ren Wang

**Affiliations:** 1 Department of Medical Laboratory Science and Biotechnology, College of Medicine, National Cheng Kung University, Tainan 701, Taiwan; e25487910@gmail.com; 2 National Institute of Infectious Diseases and Vaccinology, National Health Research Institutes, Tainan 701, Taiwan; e480216@hotmail.com (C.-H.T.); anavon31@gmail.com (Y.-F.W.); 3 Institute of Basic Medical Sciences, College of Medicine, National Cheng Kung University, Tainan 701, Taiwan; daynac_1201@hotmail.com

**Keywords:** avian influenza virus, virus-like particles, vaccine, H5N6

## Abstract

Highly pathogenic avian influenza (HPAI) clade 2.3.4.4 viruses have been reported to be the source of infections in several outbreaks in the past decades. In a previous study, we screened out a broad-spectrum virus strain, H5N6-Sichuan subtype, by using a lentiviral pseudovirus system. In this project, we aimed to investigate the potential of H5N6 virus-like particles (VLPs) serving as a broad-spectrum vaccine candidate against H5Nx viruses. We cloned the full-length M1 gene and H5, N6 genes derived from the H5N6-Sichuan into pFASTBac vector and generated the VLPs using the baculovirus-insect cell system. H5N6 VLPs were purified by sucrose gradient centrifugation, and the presence of H5, N6 and M1 proteins was verified by Western blot and SDS-PAGE. The hemagglutination titer of H5N6 VLPs after purification reached 5120 and the particle structure remained as viewed by electron microscopy. The H5N6 VLPs and 293T mammalian cell-expressed H5+N6 proteins were sent for mice immunization. Antisera against the H5+N6 protein showed 80 to 320 neutralizing antibody titers to various H5Nx pseudoviruses. In contrast, H5N6 VLPs not only elicited higher neutralizing antibody titers, ranging from 640 to 1280, but also induced higher IL-2, IL-4, IL-5, IFN-γ and TNF production, thus indicating that H5N6 VLPs may be a potential vaccine candidate for broad-spectrum H5Nx avian influenza vaccines.

## 1. Introduction

Avian influenza virus (AIV) belongs to the *Orthomyxoviridae* family, *Alphainfluenzavirus* genus, and contains eight negative-sense, single-stranded RNA segments as its genome [1]. During viral replication, reassortant genes help the AIVs to overcome the species barrier to infect humans, thus causing a major threat to public health [2]. The highly pathogenic avian influenza (HPAI) virus A/goose/Guangdong/1/96 H5N1 (Gs/GD) lineage caused global epidemics not only in migratory birds and domestic poultry but also in the human population. Taiwan had previously encountered an outbreak of HPAI clade 2.3.4.4 derived from Gs/GD lineage in poultry [3]. Clade 2.3.4.4 has evolved since 2008 and had several reassortant neuraminidase (NA) genes with N2, N3, N5, N6, and N8 variants. Phylogenetic analysis based on the sequence of hemagglutinin (HA) and M gene reveals that HPAI H5Nx viruses in October 2014 were related to two HPAI H5N8 viruses identified from wild birds in Japan. Two subsequent outbreaks in late 2014 and 2016 resulted from seven H5Nx reassortant genes that spread through Taiwan and eight novel introduced internal genes mostly derived from the gene pool of avian influenza virus circulating in wild birds in Asia [3]. In several outbreaks in past decades, it has been reported that the sources of infection were novel HPAI clade 2.3.4.4 viruses. These newly emerging, highly pathogenic H5Nx avian influenza viruses, including H5N1, H5N2, H5N3, H5N6, and H5N8, have raised great concern worldwide [4,5,6]. Taiwan also encountered the largest outbreak of avian influenza viruses in 2015, including H5N2, H5N6 and H5N8 [7]. Genetic analysis revealed that the HA and NA gene of H5N2 circulating in Taiwan before 2015 outbreaks were derived from American H5N2 (old Mexican-like lineage), and the other six gene segments were similar to local H6N1 in Taiwan [8,9]. The HA of the new epidemic strain H5N2 in 2015 belonged to clade 2.3.4.4 HPAI; therefore, the old vaccine might not be able to protect against new emerging H5N2, H5N3, H5N8 and H5N6 viruses. Thus, a new strategy of vaccine platform for rapid application is necessary.

There are three major types of influenza vaccine approved by the Food and Drug Administration in the United States, including inactivated vaccine, live-attenuated vaccine and recombinant vaccine. The manufacture of inactivated vaccine or live-attenuated vaccine is derived from a specific strain of AIV that probably confers only strain-specific immunity. In addition, inactivated vaccines and live-attenuated vaccines seldom elicit cross-protective immunity against different subtypes of AIVs. A mismatch between the available vaccines and the circulating AIV strain may contribute to high mortality and wild transmission in the avian and human population.

Virus-like particles (VLPs) are non-infectious nanoparticles made up of assembled viral proteins without viral genetic materials. In comparison to whole inactivated vaccines, VLP vaccines consisting of different combinations of antigen have the advantage to induce a broader immune response [10]. At present, highly conserved ectodomain of M2 protein (M2e) or the stalk of the HA protein have been used as the antigen for a VLP vaccine candidate for influenza viruses [11,12]. Animals vaccinated with M2e VLP have lower morbidity and viral titers, while the viral clearance has not been observed. VLP with stalk HA elicits cross-protective humoral immunity against various strains of AIVs; however, it is less immunogenic [11,13]. Several studies also posed different strategies. For example, Wu et al. fused a conserved M2e protein at the N-terminus of HA, or at the N-terminus of NA in the H5N1 VLP, to protect against heterosubtypic H1N1 and H5N1 [13]. Kang et al. have expressed two HAs derived from a different clade of AIVs to enhance robust immune response [14]; however, expression of multiple genes in one vector may also contribute to assembly instability due to the tandem repetition of similar sequences [15]. In this study, we supposed that the conformation of VLP presenting the HA and NA may be similar to the native virions that induced antibodies and will be able to target the HA, which can effectively inhibit viral replication. VLPs can be produced in several expression systems, including mammalian cells, plants, insect cells, yeast and bacteria. Since the VLPs generated from a baculovirus-infected insect cell line have advantages of high expression yield, good protein folding with glycosylation modification and ease for scale-up production, we aimed to develop a potential VLP-based vaccine by using the baculovirus-insect cell expression system.

In our previous study, we analyzed genetic evolution of HA from H5Nx viruses circulating in Taiwan. The phylogenetic analysis performed by PAUP showed that all the new H5Nx viruses isolated in Taiwan belonged to clade 2.3.4.4, except the old H5N2 circulating prior to 2015. HA and NA gene sequences derived from different avian influenza viruses, including A/Northern pintail/Washington/40964/2014 H5N2 (clade 2.3.4.4b), A/duck/Taiwan/1702004/2017 H5N6 (clade 2.3.4.4e), A/duck/Hyogo/1/2016 H5N6 (clade 2.3.4.4e), A/Sichuan/26221/2014 H5N6 (clade 2.3.4.4a), A/gyrfalcon/Washington/41088-6/2014 (H5N8) (clade 2.3.4.4b) and A/chicken/Taiwan/x37/2016 H5N8 (clade 2.3.4.4b), were used for lentiviral pseudovirus production, as described previously [16]. We further identified an H5N6-Sichuan subtype that exhibited high neutralizing titers against different subtypes [16]. A lentiviral vector-based vaccine is able to directly express the antigen of interest in target cells in vivo, which can effectively induce antigen-specific CD8+ T cell immune response [17]. Although several studies have demonstrated the protective efficacy in preclinical and clinical trials, it was still not approved to be used in humans to date. The main concern is the stability of viral integration in the host genome, which may activate the cellular protooncogene by integrated long terminal repeats (LTRs) or the transcriptional interference and suppression by LTRs [18]. Therefore, we developed a potential vaccine candidate using a virus-like particles expression system that is non-infectious and had been approved to use in humans. In order to further investigate whether H5N6 Sichuan subtype would be a potential vaccine candidate for humans, we aimed to establish a virus-like particle expression system and further evaluate the immune response elicited by VLP and 293T mammalian cell-expressed protein in mice models.

## 2. Materials and Methods

### 2.1. Cell Culture

Insect cell lines, Spodoptera frugiperda ovarian cells (Sf21) and Trichoplusia ni ovarian cells (High Five^TM^) were cultured at 28 °C in an incubator, without CO_2_ and water supplement. Sf21 cells were maintained in Grace’s Insect medium (Gibco) supplemented with 10% fetal bovine serum (FBS, Hyclone, Thermo Fisher Scientific, Carlsbad, CA, USA) for baculovirus production. For H5N6-Sichuan VLP production, High Five™ cells were cultured in serum-free HyClone™ SFM4 Insect medium (Hyclone) with 1% penicillin streptomycin mixtures (P/S).

293T cells and MDCK cells were cultured at 37 °C in an incubator with 5% CO_2_. 293T cells were maintained in DMEM supplemented with 10% FBS, 2% P/S, and 10 µM sodium pyruvate for the production of H5Nx pseudoviruses and 293T expressed H5+N6 proteins. MDCK cells were maintained in Eagle’s MEM with 1 μg/mL trypsin, 1mM sodium pyruvate, 100 U/mL penicillin and 0.1 mg/mL streptomycin for titration of pseudoviruses and neutralization assay of VLP or mammalian cell-expressed proteins immunized mice serum against H5Nx pseudoviruses.

### 2.2. Generation of Virus-like Particle Expression Plasmid

PCR products of NA were cloned into pBac-YCCRG3 plasmid containing an M1 sequence from an H7N9 subtype (pFastBac vector, gift from Dr. Yung-Chih Hu, NHRI) using KpnI and NheI restriction enzyme sites, named as pBac-YCCH7SCN6. By using BssHII and RsrII restriction enzyme sites, the amplified HA genes were cloned into the pBac-YCCH7SCN6, named as pBac3-SC. The inserted HA, NA sequences from H5N6 Sichaun subtype and M1 sequence from H7N9 subtype pBac3-SC plasmid were confirmed by Sanger sequencing. The pBac3-SC plasmid was transformed into a DH 10BacTM competent cell (Thermo Fisher Scientific) for transposition into bacmid. The colonies that contain the desired recombinant bacmid were identified by blue-white screening with 50 μg/mL kanamycin, 10 μg/mL tetracycline, 7 μg/mL gentamycin, 100 μg/mL Bluo-gal and 40 μg/mL IPTG. Sf21 cells were seeded in 6-well plates (8 × 10^5^ cells/mL) and transfected with 2.5 μg/well of pBac3-SC plasmid by TransIT-Insect transfection reagent (Mirus Bio, Madison, WI, USA), and the supernatants were collected at 72 h post-transfection. The P1 viruses were immediately amplified in confluent 6-well plates of Sf21 cells and titrated by plaque assay for further experiments. The viruses were stored at −80 °C and protected from light.

### 2.3. Production of H5N6 VLPs

The cultures were performed in 250 mL shake flasks. Each flask was inoculated with 2 × 10^5^ cells/mL with a working volume of 100 mL. After three days, when the density of High Five™ cells reached 1.6 × 10^6^ cells/mL, the cells were infected with 1 × 10^8^ to 1 × 10^9^ pfu/mL of recombinant baculovirus. The H5N6 VLPs were harvested at 3 days post-infection by centrifugation at 500× *g* (Kubota TA3780) at 4 °C for 10 min, and the yield of H5N6 VLPs was further determined by hemagglutination assay.

### 2.4. Sucrose Density Gradient for Vlp Purification

For VLPs concentration and purification, VLPs were first UV inactivated for 30 min. It was filtered with 0.45 μm filters to remove cell debris and precipitated by ultracentrifugation at 360,000× *g* (Beckman L90-K ultracentrifuge, 70Ti rotor) at 4 °C for 4 h with a 20% sucrose cushion and a drop of glycerol at the bottom of the tube. The pellets were resuspended with PBS and loaded on the top of a 20–60% sucrose gradient. After ultracentrifugation at 285,000× *g* for 2.5 h, the visible band at 45% sucrose was collected (Beckman L90-K ultracentrifuge, 40Ti rotor).

### 2.5. SDS-PAGE and Western Blot Analysis for Protein Composition of VLP

Purified H5N6 VLP samples were quantified by Qubit protein assay. Approximately 2 μg of purified H5N6 VLPs were separated on 12% SDS-PAGE. To confirm the purification efficiency of the sucrose density gradient, the SDS-PAGE gel was stained using silver staining. To detect the presence of target HA, the separated proteins on the SDS-PADE were transferred onto the PVDF membrane, blocked with 5% non-fat milk, and incubated with primary antibody (1:5000, avian flu HA antibody, Mab, Sino Biological, Beijing, China) at 4 °C overnight. The blots were then incubated with secondary antibody (1:5000, goat anti-mouse IgG, HRP-labeled, KPL) and developed using enhanced chemiluminescence (ECL, Bio-Rad, Hercules, CA, USA) substrate. All the images were captured by Amersham Imager 600.

### 2.6. Transmission Electron Microscopy for Analysis of VLP Morphology

Purified VLPs were absorbed onto a plasma-discharged copper grid. After washing with PBS, 2% phosphotungstic acid was applied for negative staining. The particles were observed under a transmission electron microscope (JEOL JEM-1400), performed by Core Facility Center, National Cheng Kung University, Taiwan.

### 2.7. Hemagglutination Assay for HA Titer of VLP

Turkey RBCs were washed with ice-cold PBS several times until the PBS remained clear after centrifugation at 500× *g* (Kubota TA3780) for 5 min. A 0.75% of turkey RBC suspension with PBS was then prepared. To measure the hemagglutination titers of VLP, VLPs were first diluted 10-fold, and a 2-fold serial dilution with PBS was performed on a 96-well plate at a total volume of 50 μL/well. Afterwards, 50 μL of 0.75 % turkey RBC suspension was added to each well and incubated at room temperature for 30 min to 1 h [19]. In the presence of VLPs, the HA on the surface of VLPs will bind to the sialic acid on the RBC, showing hemagglutination. The highest dilution titer that had shown hemagglutination was regarded as the hemagglutination titer.

### 2.8. Expression of H5-HA and N6-NA Protein in 293T Mammalian Cell System

293T cells were seeded in 15 cm dishes (0.75–0.9 × 106 /mL, 20 mL/dish). After 16 h of incubation, plasmids with pTT5 vector (pTT5-SCH5, pTT5-SCN6, Leadgene Biomedical, Inc., Tainan, Taiwan) were transfected with 15 μg of H5 or N6 plasmid DNA per dish, respectively, by Hyfect transfection reagent (Leadgene). Dishes were refreshed with cell medium (FreeStyle 293 expression medium) 24 h later. At 72 h post-transfection, supernatant was collected and centrifuged at 500× *g* to remove the cell debris. The supernatant was further precipitated via ammonium sulfate. The desired HA and NA proteins were visualized by Western blot with anti-His tag mAb (1:5000, Leadgene). The blots were then incubated with secondary antibody (1:5000, goat anti-mouse IgG, HRP-labeled, KPL) and developed using enhanced chemiluminescence (ECL, Bio-Rad, Hercules, CA, USA) substrate. All the images were captured by Amersham Imager 600.

### 2.9. Mouse Immunization

For H5N6 VLPs and H5+N6 protein antisera, 6- to 8-week-old BALB/c mice (4 mice per group) were initially immunized with the purified H5N6 VLPs and 293T cell expressed H5+N6 proteins with adjuvant (Freund’s complete adjuvant), which was performed by Leadgene Biomedical, Inc, Taiwan (IACUC-11006003). Ten mg of VLP in PBS or recombinant proteins emulsified in Complete Freund’s Adjuvant (CFA, Sigma) for priming and Incomplete Freund’s Adjuvant (IFA, Sigma) for the boost in a total of 100 μL were immunized three times at one-week intervals. Serum samples were collected by submandibular blood collection at three time-points: pre-immunization, day 14 and day 35 post-immunization (antiserum of H5+N6 proteins were not collected at day 14, since these two immunizations were performed at separate times). At day 35, mice were sacrificed and the cardiac chambers punctured for the serum sample collection. The end-point serum samples were used for neutralization assay, and the serums collected at different time-points were used to measure the induction of cytokine levels. Each serum was collected from 4 BALB/c mice and pooled. All the serum samples were aliquoted and stored at −80 °C until further use.

### 2.10. Enzyme-Linked Immunosorbent Assay (ELISA) for Specific IgG Titer

The 96-well ELISA plates were coated with 50 μL of PBS containing 1 μg/mL of H5N6 VLP or 293T expressed H5+N6 proteins, respectively. The plates were incubated at room temperature for an hour and blocked with 3% skim milk in 0.05% PBS-T. Serum samples were 100-fold and then two-fold serially diluted with blocking buffer. After being washed several times with 0.05% PBS-T, the coated plates were then incubated with 50 μL of serially diluted serum, followed by incubating with HRP-conjugated anti-mouse IgG antibodies (1:5000, Leadgene) at 37 °C for 30 min. Lastly, 50 μL of tetramethyl benzidine dihydrochloride (TMB) substrate was added in each well and stopped with 50 μL/well of 1 N H_2_SO_4_ two minutes later. The absorbance at 450 nm was measured using an ELISA plate reader (Meter-tech M965+).

### 2.11. Neutralization Assay for Neutralizing Antibody Titer

To determine the neutralizing antibody titer of H5N6 VLPs and 293T expressed H5+N6 protein immunized mouse serum against H5Nx pseudoviruses, neutralization assay was performed as previously reported [16]. MDCK cells were seeded in 24-well plates with 7.5 × 10^4^ cells/well and incubated at 37 °C for 16 h. Mouse antisera were complement inactivated at 56 °C for 30 min before neutralization assay. H5Nx pseudoviruses (5 × 10^6^–1 × 10^7^ TU/mL) were co-incubated with 2-fold serially diluted mouse serum in a 37 °C incubator for 30 min. MDCK cells were then infected with the mixture and adsorbed for 1.5 h. The serum-free DMEM medium was added after adsorption. The medium was changed to MDCK medium (Eagle’s MEM with 1 μg/mL trypsin, 1mM sodium pyruvate, 100 U/mL penicillin and 0.1 mg/mL streptomycin) after 24 h. Infected cells with fluorescence were fixed with 2% paraformaldehyde at 4 days post-infection, and the percentage of fluorescence of infected cells was measured by flow cytometer. The neutralization titers (NT50) were defined as 50% reduction of transduction unit (TU) in both duplications compared with the transduction unit of virus control.

### 2.12. Multiplex Analysis of Mouse Cytokine Productions

Th1/ Th2-related multiple cytokines were measured by BD cytometric bead array. Five beads with distinct fluorescence intensities had been coated with capture antibodies specific for IL-2, IL-4, IL-5, TNF, and IFN-γ. Antisera against H5N6 VLPs and 293T cell- expressed H5+N6 proteins were collected at different time-points and were diluted three-fold with assay diluent. Standard beads were prepared following the instruction manual. The captured beads were mixed with the serum samples and the PE detection reagents and incubated at room temperature for two hours. After washing with 1 mL of wash buffer for each tube, the tubes were centrifuged at 200× *g* for 5 min. Lastly, the pellet was resuspended with 300 μL of wash buffer, and the fluorescence was analyzed by FACS Calibur flow cytometer.

## 3. Results

### 3.1. Establishment of the H5N6 Virus-like Particles Expression System Using Baculovirus-Insect Cell System

#### 3.1.1. Construction of the VLP-Expressing Plasmid

**Figure 1:**
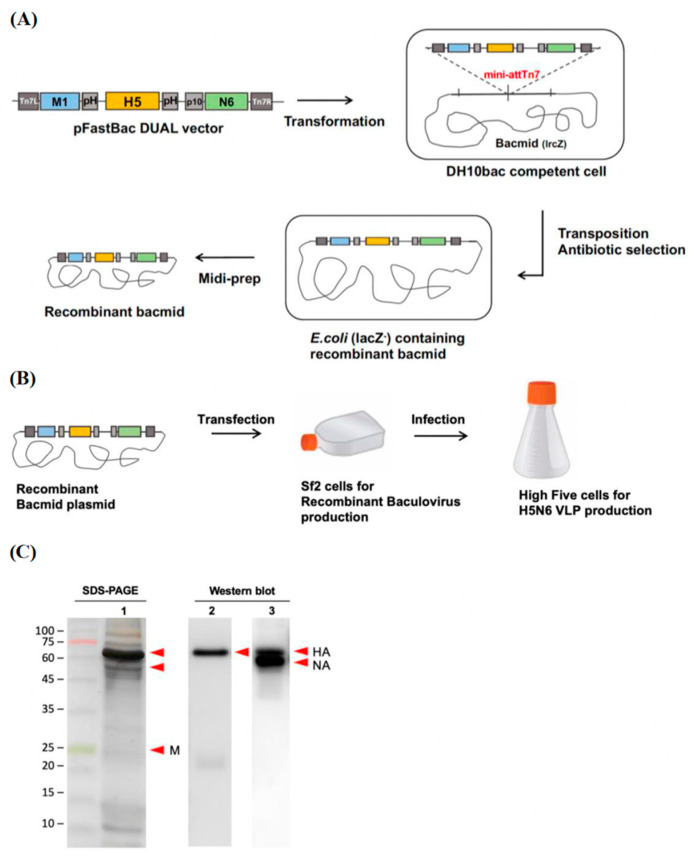
Production of H5N6 VLPs in the baculovirus expression vector system. (**A**) A schematic diagram of H5N6 recombinant bacmid construction. The recombinant bacmid carrying the hemagglutinin (HA), neuraminidase (NA) and matrix protein 1 (M1) genes derived from the H5N6 subtype was generated by mini attTn7 transposition in DH10bac competent cells. (**B**) Recombinant baculovirus collected from Sf21 cells were used to infect High Five cells for VLP production. (**C**) The expression of the HA, NA and M1 proteins on the VLPs was visualized by SDS-PAGE with silver staining (lane 1) and confirmed by Western blot using avian flu HA antibody (Sino Biological, 11048-MM06) (lane 2) and Lenti-H5N6-SC mouse antisera (lane 3) (indicated by red arrow).

In our previous study, we established a lentiviral pseudovirus system, which is able to rapidly screen out the broad-spectrum virus strain during outbreaks and examine vaccine immunogenicity. Through the neutralization assay of the generated avian influenza H5 subtypes pseudoviruses antisera, we found that H5N6-Sichuan subtype antisera exhibited better cross-protectivity among H5N2, H5N6 and H5N8 subtypes [16]. In order to further investigate whether H5N6-Sichuan strain could be a potential vaccine candidate, we first generated the virus-like particles derived from H5N6-Sichuan strain by using a Bac-to-Bac baculovirus expression system. The full-length HA and NA derived from H5N6-Sichuan were PCR amplified and cloned into pFASTBac vector. The plasmid was then transformed into DH 10BacTM competent cells, which contain a bacmid DNA. Using mini-attTn7 site-specific transposition, the inserted foreign genes will disrupt the LacZα gene, and the recombinant bacmid containing the desired insert can be identified by blue-white selection (Figure 1A).

#### 3.1.2. Generation of the H5N6 Virus-like Particles from Sf21 and High Five Cells

After we confirmed the inserted gene in the recombinant bacmid by sequencing, the recombinant bacmid were amplified in LB broth and extracted with Qiagen plasmid midi kit. Previously, Lai et al. had demonstrated that Sf21 cells are suitable for a large amount of baculovirus production, while the VLPs are more easily generated by High Five™ cells [20]. Therefore, we transfected the recombinant bacmid with TransIT transfection reagent into Sf21 cells cultured in an adherent flask to generate the recombinant baculovirus. After three days of culture, the recombinant baculoviruses budded into the supernatant and were immediately propagated by infecting High Five™ cells. High Five™ cells were infected with MOI 1 of recombinant baculovirus from the above culture of Sf21 cells (Figure 1B). SDS-PAGE was first performed to analyze the production of the H5N6 VLPs. The results showed the presence of HA (64.08 kDa) and NA (52.4 kDa) proteins, followed by confirmation by Western blot with specific antibodies of avian flu H5N1 HA antibody and mouse antisera against lenti-H5N6 Sichuan pseudoviruses (Figure 1C).

#### 3.1.3. Precipitation and Purification of H5N6 VLP by Sucrose Density Gradient

**Figure 2:**
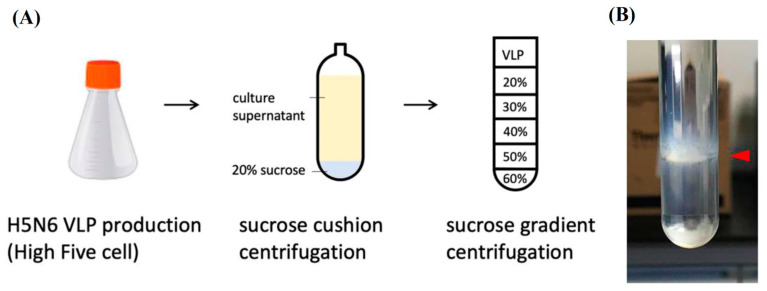
Purification of H5N6 VLPs by sucrose gradient. (**A**) Purification steps for H5N6 VLPs purification. The H5N6 VLPs were precipitated by a 20% sucrose cushion first and further purified by a 20–60% sucrose density gradient. (**B**) A visible band at 45% of sucrose fraction indicated the purified H5N6 VLPs.

In order to investigate whether the H5N6 VLPs induced immunogenicity, purification and concentration of H5N6 VLPs is needed for mice immunization. Previous studies have shown that norovirus capsid VLPs purified by sucrose density gradient obtained high yield and were more stable compared to other methods [21]. At day three post-infection, 250 mL of culture supernatant was clarified and precipitated by low-speed centrifugation with a 20% sucrose cushion and a drop of glycerol at the bottom of the tube. The pellet was resuspended with 1.5 mL of PBS and loaded onto the 20–60% sucrose density gradient. After ultracentrifuge, a visible band at 45% sucrose was observed, which indicated the purified H5N6 VLPs. Fractions from 15%, 25%, 35% and 45% sucrose were collected from the bottom for further analysis of the purification efficiency (Figure 2A,B).

### 3.2. Characterization of H5N6 VLPs Properties

#### 3.2.1. The Protein Composition of the H5N6 VLPs

**Figure 3:**
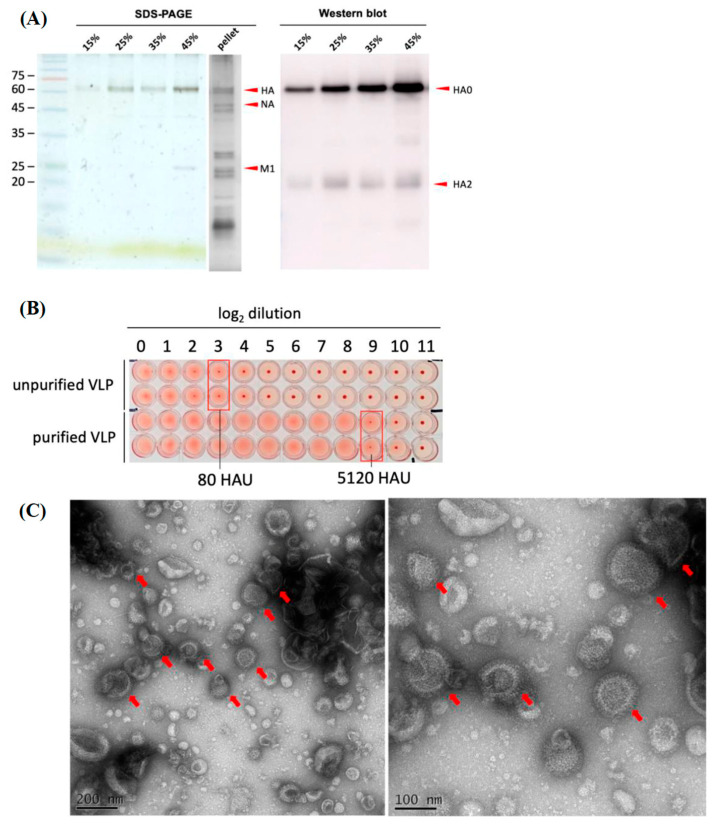
Protein expression, hemagglutination and structure of H5N6 VLP after sucrose gradient purification. (**A**) The expression of the HA, NA and M1 proteins on the VLPs at different percentages of sucrose fraction was visualized by SDS-PAGE with silver staining and confirmed by Western blot using avian flu HA antibody, Mab (Sino Biological, 11048-MM06). The pellet of H5N6 VLPs was precipitated by a 20% sucrose cushion. (**B**) Comparison of hemagglutination activity between unpurified and purified VLPs (at 45% sucrose fraction) by hemagglutination assay. The serum sample started from a 10-fold dilution. (**C**) The H5N6 VLPs after sucrose gradient purification were imaged using transmission electron microscopy by negative staining. Scale bars, Left: 200 nm, Right: 100 nm.

In order to verify whether the major immune epitope, HA proteins, still remained in the H5N6 VLPs after purification, we detected the proteins by Western blot using avian flu anti-HA antibody. SDS-PAGE with silver stain was also performed to examine the purity of H5N6 VLPs. The results showed that the fraction of 45% sucrose not only had the majority of the HA but also the NA and M1 proteins; furthermore, other proteins derived from the High Five cells were mostly removed after purification (Figure 3A).

#### 3.2.2. The Hemagglutination Property of the H5N6 VLPs Remained

Hemagglutinin glycoprotein was reported as the receptor-binding protein of the influenza virus. In order to examine whether sucrose gradient purification affects the hemagglutination property, hemagglutination assay was performed. Approximately 250 mL of VLPs in the culture supernatant were precipitated and resuspended with 4 mL of PBS. Then, 4 mL of concentrated VLP was purified and the final volume of purified VLP was about 1 mL. The result indicates that the hemagglutination property of H5N6 VLPs purified from sucrose gradient ultracentrifugation can reach 5120 HAU/ 50 μL. In contrast, the HA titer of H5N6 VLPs before concentration and purification was only 80 HAU/ 50 μL (Figure 3B).

#### 3.2.3. The Morphology of the H5N6 VLPs Remained Structurally Intact after Purification

To examine the formation of the H5N6 VLP, we directly visualized the particles using negative stain by transmission electron microscopy. The results showed that H5N6 VLPs were successfully prepared with morphology that resembled the native virions without genetic materials. The particles were homogenous and approximately 110 nm in size (Figure 3C). These data suggested that VLPs derived from H5N6-Sichuan strain can be successfully generated through a baculovirus expression system. After being concentrated and purified by a sucrose cushion and density gradient, H5N6 VLPs maintained a functional hemagglutination property and were structurally intact.

### 3.3. Generation of 293T Mammalian Cell Expressed H5+N6 Protein

**Figure 4:**
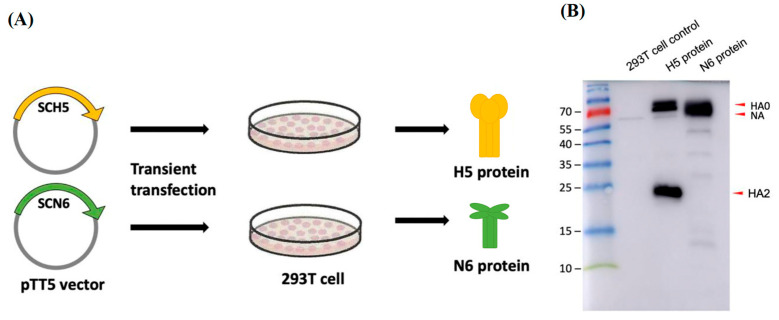
Expression of HA and NA proteins derived from H5N6 Sichuan strain in 293T mammalian cells. (**A**) Schematic diagram of mammalian cell expressed H5-HA and N6-NA proteins. The HA and NA genes were cloned into a pTT5 protein expression vector and transfected into 293T cells. After 4 days of culture, the H5 and N6 proteins were collected from the culture supernatant. (**B**) The presence of H5 (62 kDa) and N6 (56.4 kDa) proteins was verified by anti-His tag antibody in Western blot.

To investigate whether the virus-like structure of VLPs is helpful to induce superior immunogenicity, we also used 293T mammalian cells to express H5 HA and N6 NA genes derived from the H5N6 Sichuan strain for comparison. The clones of HA and NA constructed in pTT5 expression vector were performed by Leadgene Biomedical, Inc. The plasmids were separately transfected into a different dish of 293T cells, the supernatant containing the desired H5 HA and N6 NA proteins was collected at day 4 post-transfection and the proteins were precipitated by ammonium sulfate (Figure 4A). The H5 and N6 proteins were observed by Western blotting with anti-His tag antibody and pooled for mice immunization (Figure 4B). 

### 3.4. Investigation of the Immune Response Elicited by the H5N6 VLPs and 293T Cell-Expressed H5+N6 Protein

#### 3.4.1. Specific Total IgG Antibodies Were Produced in Immunized Mice

**Figure 5:**
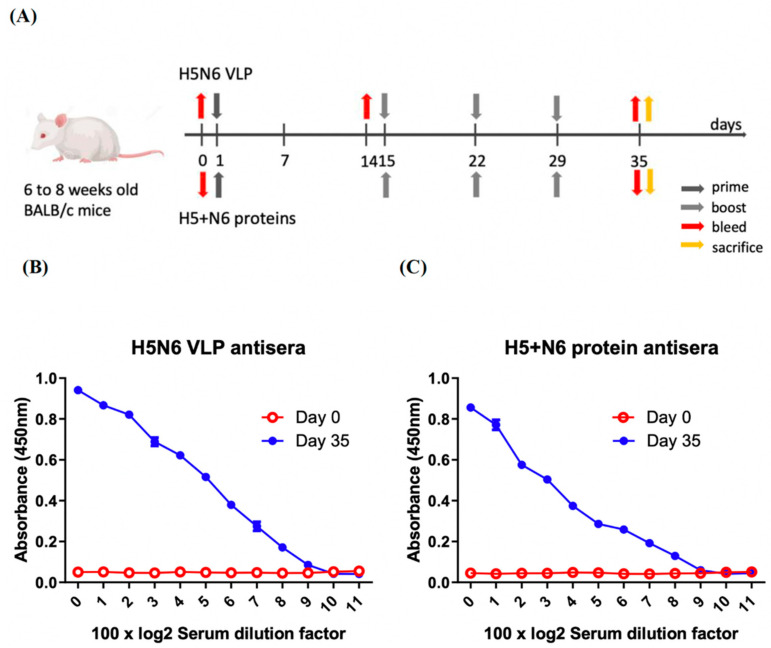
Specific IgG antibody responses in mice immunized with H5N6 VLP or 293T cell-expressed H5+N6 protein. (**A**) Experimental design for mice immunization (n = 4 per group). The mice were immunized and boosted at one-week intervals after 14 days post-immunization (d.p.i). Serum samples were collected by submandibular blood collection at pre-immunization (Day 0), day 14 and day 35 post-immunization (Day 35). The total specific IgG level at Day 0 and Day 35 in H5N6 VLP antiserum (**B**) and H5+N6 protein antiserum (**C**) against their immunized antigens were analyzed by ELISA.

Groups of BALB/c mice were immunized by intraperitoneal route (i.p) with purified H5N6 VLPs or 293T cell expressed H5+N6 proteins. Serum samples were collected at pre-immunization and day 35 post-immunization (Figure 5A). The level of total IgG was measured by ELISA. The results showed that pre-immunized antisera had no specific IgG antibody against H5N6 VLPs and 293T cell-expressed H5+N6 proteins (Figure 5B,C). At day 35 post-immunization, IgG antibody end-point titers against their immunized antigens were both 100 × 2^10^ in H5N6 VLPs and 293T cell-expressed H5+N6 proteins antisera, suggesting that mice immunized with H5N6 VLPs and 293T cell-expressed H5+N6 proteins successfully generated the IgG antibodies against the antigens (Figure 5B,C).

#### 3.4.2. Neutralization Antibodies Were Higher in VLP Immunized Mice

To compare the immunogenicity induced by H5N6 VLPs and 293T cell-expressed H5+N6 protein in mice, after three rounds of immunization with each immunogen formulated with adjuvant, mice were sacrificed and serum samples were collected by submandibular blood collection. Neutralization titers of antisera were distinguished by over 50% reduction of virus transduction in duplicate. The results demonstrated that H5+N6 protein antisera only elicited partial cross-reactive antibodies, ranging from 80 to 320 against different subtypes of avian influenza pseudoviruses, including H5N2, H5N6 and H5N8. In contrast, H5N6 VLPs antisera exhibited higher neutralizing antibody titers, between 640 to 1280 (Table 1). These findings indicated that H5N6 VLP can induce higher neutralizing antibodies than 293T cell-expressed H5+N6 proteins, suggesting that the structure of VLP is helpful in displaying the immune epitope on the surface, which might result in better cross-reactive neutralizing antibodies.

#### 3.4.3. Cytokine Production

**Figure 6:**
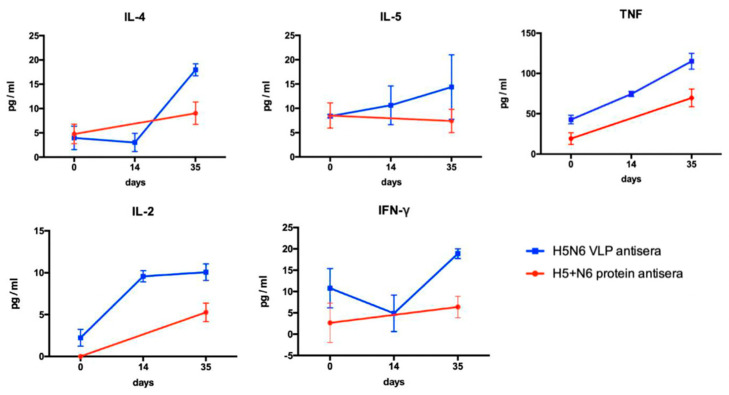
Cytokine profiles of mice immunized with H5N6 VLPs or 293T cell-expressed H5+N6 proteins. Antisera of vaccinated mice were collected at different timepoints: pre-immunization, day 14 and day 35 post-immunization The cytokines (IL-2, IL-4, IL-5, IFN-γ, and TNF) in antisera were measured by cytometric bead array (CBA) multiplex assay. Serum samples collected at each time-point were measured in duplicates.

Th1 and Th2 cells play an important role in adaptive immunity. After immunization, Th1 cells secrete IFN-γ, TNF and IL-2 to mediate cytotoxic T cell and opsonizing antibodies. Th2 cells stimulate the humoral immune response by secreted IL-4 and IL-5, which can regulate B cell proliferation and induce antibody production. We measured the levels of multiple cytokines to investigate the 293T cell-expressed H5+N6 protein or H5N6 VLPs induced immune response. H5+N6 protein and H5N6 VLPs antisera were collected at preimmunization, day 14 (H5N6 VLP only) and day 35 post-immunization. The results demonstrated that H5N6 VLPs can induce a higher production of Th1- and Th2-related cytokines (Figure 6). These data suggest that H5N6 VLPs have the advantage of particle formation that can display HA protein on the surface, which stimulated higher levels of cytokine production to eventually help to generate more neutralizing antibodies.

## 4. Discussion

Highly pathogenic avian influenza epidemics pose serious threats to socioeconomic and animal health. In the past decades, multiple outbreaks of H5 HPAI viruses have occurred, especially in Asian countries, including H5N1 in China and Vietnam, H5N2 in North America and Taiwan, H5N6 in China, Laos and Vietnam, and H5N8 in China, Europe, Japan, North America, South Korea and Taiwan [22]. Recently, reinvasion of H5N8 subtype viruses in Japan resulted in the cull of 1.9 million birds in October 2020 [23]. In order to develop an effective vaccine with robust protectivity for epidemic prevention, we established a VLP expression system and generated VLPs derived from a broad-spectrum virus strain, H5N6-Sichuan subtype, as a vaccine candidate. We compared the immune response elicited by H5N6 VLPs and 293T mammalian cell-expressed H5+N6 proteins. The results showed that Th1- and Th2-related cytokines were induced by both antigens, while the level of cytokines was higher in H5N6 VLPs antisera, which resulted in a higher titer of cross-reactive neutralizing antibodies. Combined with the lentiviral–pseudovirus system, we can efficiently produce a large amount of VLPs carrying HA from a broad-spectrum virus strain during outbreaks, which may be helpful to the poultry industry and public health in the human population.

Evaluation of humoral and cellular immunity is an essential aspect to examine the effectiveness of vaccination. CD4+ T cells are activated via the secretion of Th2-related cytokines (IL-4, IL-5) to stimulate B cells for the production of antibodies to prevent viral infection. CD8+ T cells, stimulated by Th1-related cytokines, are responsible for killing the infected cells. ELISA was used to detect all the binding antibodies in the antisera. In our results, H5N6 VLPs induced a higher production of Th1- and Th2-related cytokines that lead to higher titers of neutralizing antibody in equal amounts of specific IgG level, measured by ELISA, in H5N6 VLPs and 293T cell-expressed H5+N6 protein antisera. We suggested that H5N6 VLP, with the advantage of displaying the immune epitope on the particle, exhibited a higher titer of neutralizing antibody. A large portion of specific IgG antibodies detected in 293T cell-expressed H5+N6 protein antisera may be the non-neutralizing antibody. Although non-neutralizing antibody is not able to prevent viral infection, it may still contribute to antiviral protection through other antibody-mediated effects, such as antibody-dependent cellular cytotoxicity (ADCC), antibody-dependent cellular phagocytosis (ADCP) or antibody-mediated complement-dependent cytotoxicity (CDC) [24].

In our findings, antisera against H5N6 VLP exhibited a neutralizing antibody titer of 1280 against both Lenti-H5N6-Sichuan and Lenti-H5N8-Washington subtypes, which can be correlated to their close proximity on the antigenic cartography of avian influenza virus H5Nx pseudoviruses [16]. In addition to generating broad-spectrum VLPs with single HA as vaccine candidate, scientists had also proposed some other strategies for VLP-based vaccine development. Kang et al. exploited multi-clade VLPs carrying two HAs derived from two clades, clade 2.3.2.1c and clade 2.3.4.4c, within a single vector, and found that multi-clade VLPs not only show high productivity but also induced effective antibodies [14]. In contrast to variable surface antigens of HA and NA, the ion channel protein, Matrix protein 2 ectodomain (M2e), represents an alternative antigen target for AVI vaccine, since M2e protein has a relatively conserved sequence and plays a critical role in virus assembly and budding during replication. Mice intranasally immunized with a pandemic live attenuated vaccine supplemented with M2e-VLPs were prevented from weight loss and had low viral titers when challenged with heterosubtypic virus, indicating that the combination of split vaccine with M2e-VLPs provides cross protection by eliciting IgG, IgA antibodies as well as M2e-induced T cell response [25]. Apart from targeting the conserved epitope, Carter et al. have reported a new method, computationally optimized broadly reactive antigens (COBRA), in order to overcome the deficiency of weak protectivity induced by M2e or NP antigens when challenged with high-dose viruses. They provided a consensus HA sequence using multiple rounds of consensus building to generate a candidate antigen and expressed the COBRA HA proteins on VLP for broadly reactive, functional antibody responses [26].

Although vaccination is helpful to control the spread of AIV, even highly immunogenic AIV strains were not able to elicit sufficient immune response without appropriate adjuvants. Previous studies had evaluated the immune response between different adjuvants for AIV vaccine in poultry [27,28]. In this study, we vaccinated the mice model with addition of complete Freund’s adjuvant (CFA) and incomplete Freund’s adjuvant (IFA) to gain a strong and long-lasting immune response. Both of them are a water-in-oil emulsions mixture prepared at a ratio of 85% mineral oil and 15% emulsifier, as described by Stone et al. [29]. The difference between IFA and CFA is that CFA contains additional heat-killed mycobacteria (Mycobacterium tuberculosis), which dramatically enhanced the immune responses by trafficking the macrophage and other immune cells to the inoculation site. In general, CFA was used with initial injection for the potentiation of T helper cells that leads to the production of immunoglobulins and effector T cells. IFA is for subsequent immunization following a strong Th2 immune response through the formation of a depot at the injection site and the stimulation of antibody producing plasma cells. The cytokine responses to H5N6-VLPs and H5N6 protein were increased to a certain degree when induced by CFA and IFA adjuvants. Since the immune response to CFA, IFA and commercial oil-based adjuvants such as Montanide ISA 70VG, 71VG, 760VG, GEL01, etc., were in a large variation [28], the use of CFA and IFA might not be able to exactly mimic the clinical situations. We used CFA and IFA for the purpose of evaluating immune response between H5N6-VLPs and H5N6 protein, while ISA 71 VG and Montanide ISA VG70 oil adjuvant might be a better choice to compare the results with others [14,30].

Taiwan has not approved the use of AIV vaccine to date. The main concerns are that a higher virus mutation rate may result in gene reassortment triggered by inactivated and live-attenuated vaccine, and there may be asymptomatic transmission between vaccinated animals. The distinct host range of AIVs also makes it difficult to raise the population immunity. Although there is still a wide spectrum of opinions about the issue of whether vaccination can actually control the spread of virus, Walker et al. have used the Bayesian Markov Chain Monte Carlo (MCMC) data augmentation technique to evaluate the impact of the vaccination in northern Vietnam. The results demonstrated a significant decrease in the transmissibility of infection following vaccination and the wave of outbreaks were less intense [31]. They suggested that vaccination is helpful to reduce the transmission of infection. In conclusion, based on our results, we suggest that H5N6-VLPs might be a potential vaccine candidate, since they induced potent humoral and cellular immune response to produce high levels of cross-reactive neutralizing antibodies and cytokines. The strategy of generating a broad-spectrum VLP with single HA as a vaccine candidate may be feasible during urgent AIV outbreaks.

## Figures and Tables

**Table 1:** Neutralizing antibody titers of H5N6 VLP-induced antisera and 293T cell expressed H5+N6 protein-induced antisera against various H5Nx pseudoviruses.

Antisera	Pseudoviruses Tested (Neutralizing Antibody Titer)					
	Lenti-H5N2 Washington	Lenti-H5N6 TW17	Lenti-H5N6 Hyogo	Lenti-H5N6 Sichuan	Lenti-H5N8 Washington	Lenti-H5N8 TWX37
H5N6 VLP	640	640	640	1280	1280	640
H5+N6 proteins	80	80	80	320	320	80

## Data Availability

The data that support the findings of this study are available within the article.
